# Whole-genome selection signature differences between Chaohu and Ji’an red ducks

**DOI:** 10.1186/s12864-024-10339-6

**Published:** 2024-05-27

**Authors:** Ruiyi Lin, Huihuang Li, Weilong Lin, Fan Yang, Xinguo Bao, Chengfu Pan, Lianjie Lai, Weimin Lin

**Affiliations:** College of Animal Sciences, Fujian Agriculture and Forestry University, Fuzhou, 350002 People’s Republic of China

**Keywords:** indigenous duck, population structure, genetic diversity, selection signatures

## Abstract

Assessing the genetic structure of local varieties and understanding their genetic data are crucial for effective management and preservation. However, the genetic differences among local breeds require further explanation. To enhance our understanding of their population structure and genetic diversity, we conducted a genome-wide comparative study of Chaohu and Ji’an Red ducks using genome sequence and restriction site-associated DNA sequencing technology. Our analysis revealed a distinct genetic distinction between the two breeds, leading to divided groups. The phylogenetic tree for Chaohu duck displayed two branches, potentially indicating minimal impact from artificial selection. Additionally, our ROH (runs of homozygosity) analysis revealed that Chaohu ducks had a lower average inbreeding coefficient than Ji’an Red ducks. We identified several genomic regions with high genetic similarity in these indigenous duck breeds. By conducting a selective sweep analysis, we identified 574 candidate genes associated with muscle growth (*BMP2*, *ITGA8*, *MYLK*, and *PTCH1*), fat deposits (*ELOVL1* and *HACD2*), and pigmentation (*ASIP* and *LOC101797494*). These results offer valuable insights for the further enhancement and conservation of Chinese indigenous duck breeds.

**Supplementary Information:**

The online version contains supplementary material available at 10.1186/s12864-024-10339-6.

## Introduction

Ducks play a crucial role in enhancing the ecological diversity of species in regions with rich breeding potential. Over time, ducks have been selectively bred for meat and eggs, resulting in significant variations in shape, productivity, and reproductive rates. Meat ducks are known for their fast growth rate and high meat production, whereas egg-laying ducks are valued for their exceptional laying performance and durability, although they tend to be smaller in size [1]. The domestication of indigenous ducks in China dates back 2000 years, with recent research indicating their evolution from *Anas platyrhynchos* and *Anas zonorhyncha* [2]. The substantial improvements in production performance and phenotypic differences between domestic ducks and mallards can be attributed to directional selection and domestication to meet market demands [3]; for instance, there are significant differences in their skeletal systems [4], as well as differences in the production performance of meat and egg-laying ducks. Nonetheless, the specific details regarding the timing and process of differentiation between meat and egg-laying ducks in Chinese indigenous breeds remain unclear, and the genetic mechanisms underlying the differences in production performance remain poorly understood.

Natural pigments accumulate in the tissues and organs of animals, playing roles in various biological processes and regulating organism metabolism. In birds, plumage color is primarily determined by melanin, carotenoids, and porphyrin pigments [5]. Plumage color serves primarily for protection [6], mate selection [7], signal recognition [8] and detoxification [9]. The genes *MITF* and *EDNRB2* [10] regulate black and white plumage colouration in ducks, while red plumage colors are less studied. Recent studies have revealed complex differences in feather colouring between geographic locations [11], feather growth regions [12], and genders [13]. Some of these factors also have a relatively high heritability.

The Middle-lower Yangtze Plain is a middle and lower coastal strip plain below the Three Gorges of the Yangtze River in China. Most of this area falls within the northern subtropics, with a small portion at the northern edge of the meso-subtropics. The plain has an average elevation of 5–100 m and is known for its abundant water resources. The region’s average annual temperature ranges from 14 to 18 degrees Celsius, creating excellent ecological conditions for waterfowl rearing. Consequently, a diverse range of local duck breeds have thrived in this area. For our study, we selected two representative breeds to investigate the genetic differences resulting from direct selection. Chaohu duck (CH), a meat and egg variety of mallards, originates from Lujiang County in Anhui Province (31°23′ N, 117°29′ E). These ducks exhibit strong adaptability, tolerance to roughage, and high disease resistance. The average weight of the males was 2.5 kg, while that of the females was approximately 2 kg. Ji’an Red duck (JA), another meat and egg variety of the mallard, originated in Suichuan County, Ji’an City, Jiangxi Province (26°33′ N, 114°5′ E). These ducks have a short and round body shape, moderate size, and a red coat color, making them ideal for the processing of dried salted duck meat. The average weight of the males is 1.5 kg, while females weigh around 1.335 kg.

The decreasing cost of high-throughput sequencing and continuous advancements in sequencing technologies have led to an increase in genomic-level analyses of various species. Previous studies on ducks have utilized selective sweep analysis to identify genes associated with feed conversion rate [14], lipid metabolism, muscle function [15], and feather color differentiation [10, 16]. Selective sweep analyses in other species have identified numerous genes that are beneficial for production efficiency and species conservation [17–19]. Furthermore, analysing inbreeding relationships between populations can aid in optimizing conservation and breeding plans for local breeds [20]. In the analysis of runs of homozygosity (ROH), genomic regions with high levels of inbreeding are referred to as ROH islands, which may serve as indicators of positive selection, possibly arising due to linkage disequilibrium. Previous studies on other species have used continuous ROH analysis to quantify ROH islands and uncover numerous genomic regions influenced by environmental changes and human interventions [20]. However, relevant analyses of indigenous ducks in China still have substantial gaps.

The purpose of this study was to investigate the differences between CH and JA after selection in different environments. We aimed to describe the genetic relationships between these two indigenous duck populations in China using principal component analysis, structure analysis, and phylogenetic tree analysis. ROH analysis is employed to evaluate valuable genomic information related to inbreeding. Based on this analysis, we identified and analysed the fixed mutant regions in the two indigenous ducks through selective sweep analysis. The averaged population differentiation index (Fst) is calculated, and the cross-population composite likelihood ratio test (XP-CLR) is conducted to reduce false-positive rates in Fst calculations [21]. The findings of this study can contribute to a better understanding of the genomic structure of the Middle-lower Yangtze Plain indigenous ducks and provide insights into potential selection signs in ROH islands. These findings are useful for developing conservation strategies for geneticists and breeders, as well as improvement schemes for the future.

## Results

### RAD sequencing of Chaohu and Ji’an red ducks

Following sequencing, the average amount of raw pair-reads per sample was 1.5 Gb. Reads containing ≥10% unidentified nucleotides and > 50% of bases with a Phred quality score below 20 were discarded. The remaining reads were aligned to the barcode adapter. Finally, clean pairs of reads were obtained after quality filtering. The sequence analysis revealed that the quality range of Q20 was between 96.00 and 96.64%, with a mean value of 96.26%. Meanwhile, the quality range of Q30 was between 89.11 and 90.62%, with a mean value of 89.72%. Genome comparison revealed a total of 4,566,782 SNPs, 150,805 insertions, and 211,058 deletions.

### Population structure analysis

**Fig. 1:**
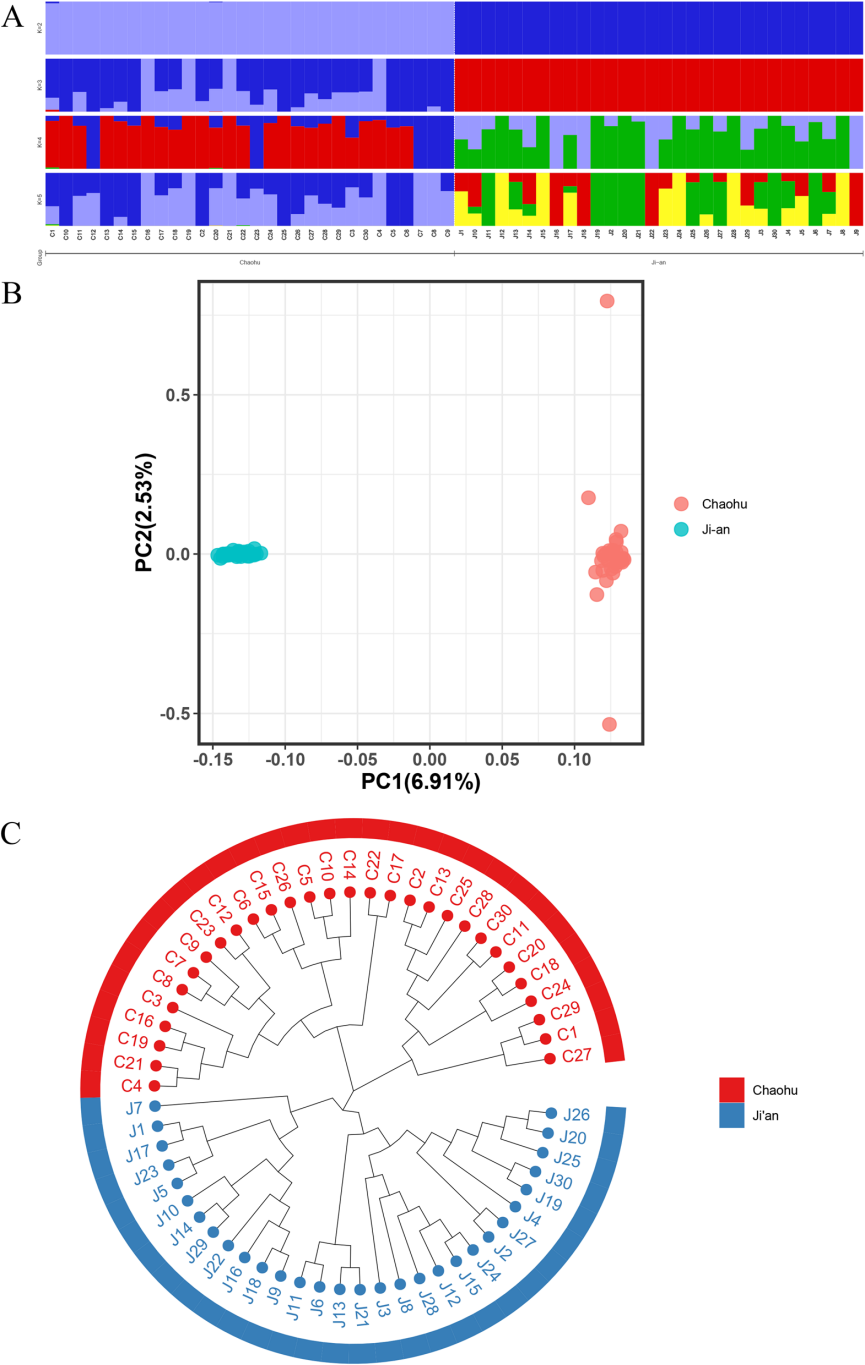
Analysis of population variation and structure analysis of duck breeds. **A**: Population structure of two ducks. **B**: Principal component analysis (PCA) for the first and second components. **C**: Phylogenetic trees showing the genetic structure of the 60 indigenous duck individuals

To determine the proportions of different ancestry groups, we used ADMIXTURE software for optimal clustering analysis. Additionally, we examined population structure based on cross-validation (CV) error result. The results of the CV error indicated that 2 clusters were optimal (Fig. S1). The population structure analysis was conducted assuming ancestral populations with parameter K (Fig. 1A). When K = 2, the separation between CH and JA was more pronounced, which aligns with the results for the samples we collected. However, when K = 3, CH exhibited mixed ancestry with a notable observation that the C1 individual displayed 3.57% JA ancestry. This finding suggests that the genealogy of CH is more complex than that of JA, potentially due to JA’s increased tendency for inbreeding during the breeding process.

To explore the genetic background of the two local duck breeds, genome-wide SNP data were subjected to principal component analysis (PCA). The analysis revealed a distinct genetic structure, with each breed forming a separate cluster (Fig. 1B). In the first component, CH and JA were clearly separated into distinct clusters. For the second and third components (Fig. S2), the JA group exhibited greater concentrations than did the CH group, further supporting the complexity of the genealogy of CH in comparison to that of JA. Additionally, the second and third components displayed numerous overlapping points between CH and JA, implying the possibility of a shared ancestor between the two breeds.

Phylogenetic trees provide valuable insights into the genetic relationships among populations. The sampling process was repeated 1000 times using the Kimura two-parameter model to ensure the robustness of the phylogenetic tree results. Samples with high sequence similarity were aggregated to construct a neighbor-joining (NJ) tree, whose topology was supported by a bootstrap value of 80% or greater. This high bootstrap value indicates the high reliability of the constructed phylogenetic tree. The results (displayed in Fig. 1C) demonstrate that all individuals of the same variety clustered together, highlighting significant genetic differentiation between CH and JA. Furthermore, the NJ tree was divided into three branches, with JA forming an independent branch and CH being divided into two branches. This division may imply that CH was subject to less artificial selection than was JA.

### Runs of homozygosity detection and analysis

**Fig. 2:**
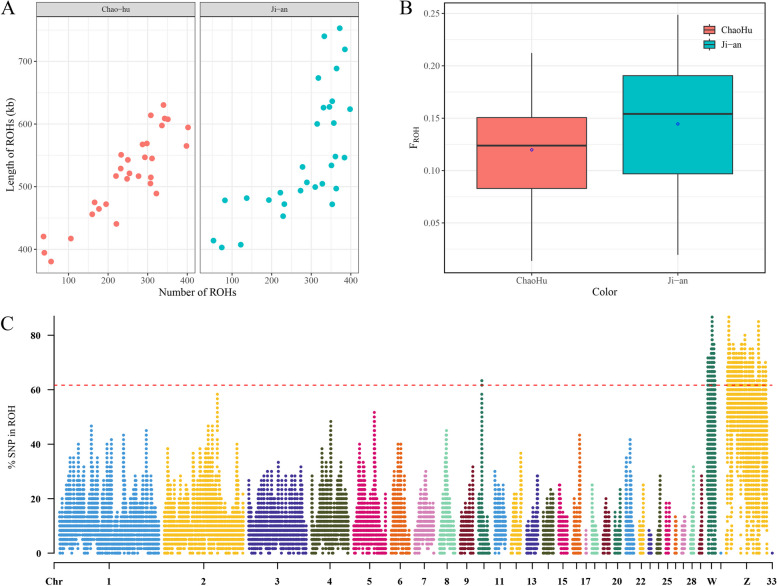
Genome-wide scan for ROH. **A**: Number of ROH per breed (X-axis) and the total ROH length of each animal (Y-axis). **B**: Box plot of the inbreeding coefficients inferred from ROH (FROH) for duck breeds. **C**: Manhattan plot of the incidence of each SNP in the runs of homozygosity among the duck breeds

A total of 15,981 ROHs were detected in both species. Among these, 7475 (46.77%) ROHs with an average length of 134.88 MB were identified in CH, while 8506 (53.23%) ROHs with an average length of 162.72 MB were identified in JA. The number and total length of ROHs (Fig. 2A) were smaller in CH than in JA.

In this study, we utilized ROH data to assess the average inbreeding coefficient among two duck populations (Fig. 2B). JA exhibited a greater mean inbreeding coefficient (F_ROH_ = 0.1445) than CH (F_ROH_ = 0.1198). At the individual level, JA had the highest genomic inbreeding coefficient (ROH_MAX_ = 0.249), while CH had the lowest (ROH_MIN_ = 0.014). These findings indicate that JA has a greater degree of inbreeding than CH. We calculated the proportion of SNPs in ROH segments, sorted the values, and established the top 1% as a threshold for identifying ROH islands (Fig. 2C, Table S1).

### Selective sweep screening and analysis

**Fig. 3:**
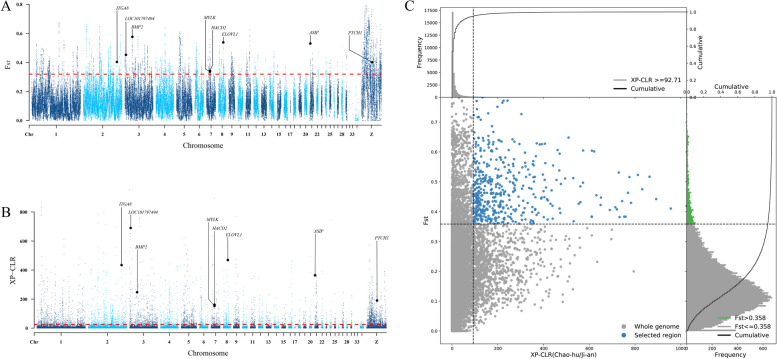
Select Sweep Analysis. **A**: Significant loci from Fst screening. **B**: Significant loci from XP-CLR. **C**: Duplicate loci from Fst and XP-CLR screening

We integrated the SNP data and conducted screening using the Fst method (Fig. 3A, Table S2). Subsequently, we employed the XP-CLR method (Fig. 3B, Table S3) with CH as the reference population and JA as the selected population. By combining the results of both screening methods, we identified a total of 574 selected genes (Fig. 3C, Table S4).

Gene Ontology (GO) and Kyoto Encyclopedia of Genes and Genomes (KEGG) analyses were then performed on the 574 genes. The results revealed showed that 259 GO terms were enriched in the GO functional enrichment analysis, and 104 pathways were enriched in the KEGG pathway enrichment analysis. The majority of the genes were associated with developmental process (GO:0032502), growth (GO:0040007), pigmentation (GO:0043473) (Fig. S3, Table S5) and fatty acid elongation pathway (ko00062) (Fig. S4, Table S5). Several candidate genes related to muscle growth were identified, such as *BMP2*, *ITGA8*, *MYLK*, and *PTCH1*. Additionally, the following genes associated with fat deposits were detected: *ELOVL1* and *HACD2*. Furthermore, we identified *ASIP*, a gene related to pigmentation.

## Discussion

Detecting the imprints of positive selection on a species’ genome through changes in gene polymorphisms enables the exploration of genetic mutations resulting from adaptive evolution. It also allows the detection of genetic mutations arising from differentiation due to spatial and temporal segregation. Previous studies on ducks have relied mainly on genome-wide association analyses, with only a few utilizing whole-genome resequencing [14]. In the present study, we employed whole-genome restriction site-associated DNA sequencing (RAD-seq) to comparatively analyse two duck breeds based on SNP markers in their genomes. Our objective was to characterize the genetic diversity and population structure of both breeds. The results of the structural analysis and PCA were consistent with the phylogenetic tree, providing evidence that the two duck species are independent ecological populations. Furthermore, the ROH analysis revealed a high degree of genetic diversity in both the CH and JA breeds.

This study involved the sequencing and analysis of 60 ducks to examine their population structure via structure analysis, principal component analysis, and phylogenetic tree analysis. Based on the structural analysis and PCA, the ducks were classified into distinct two groups, which was further supported by the division of the phylogenetic tree into two sub-branches. These findings indicate the divergence of the two duck species. The CH breed primarily originates from Chaohu city, Hefei city, Anhui Province, China, and is highly esteemed. Historical records indicate that this breed has been raised for more than 200 years. The JA breed, on the other hand, is mainly found in Ji’an County, Ji’an City, Jiangxi Province, and is known for its exceptional duck production capabilities. Historical records show that the JA breed has been raised for more than 150 years, with local residents actively selecting and refining the breed through artificial selection.

The analysis of ROH is a commonly used method for assessing the extent of inbreeding in animal populations and identifying traits associated with population selection [20]. It is deemed one of the most effective methods for detecting inbreeding [22, 23]. In this study, we identified 56 ROH islands located on chromosomes 10, W, and Z, providing further insights into the genetic landscape of the duck breeds.

Fst analysis is commonly used to identify loci with high levels of differentiation. However, these loci may not always be directly associated with the traits or environmental factors of the target species. To complement the Fst approach, the XP-CLR method was used to model frequency differences in shared alleles between populations. By simulating genetic drift under neutral conditions using Brownian motion and estimating the impact of selection clearance on nearby SNPs using a deterministic model, XP-CLR is widely used for detecting natural selection. Previous studies on livestock and poultry have shown that combining XP-CLR with Fst can effectively reduce the false-positive rate of Fst, thereby increasing the reliability of the findings [19, 24, 25]. In our study, we conducted joint screening and identified a total of 574 genes with significant differences, including 180 non-coding RNA genes and 394 protein-coding genes. Among the protein-coding genes, we identified specific genes associated with adipose tissue accumulation, myogenesis, and color divergence in feathers.

GO annotations indicated that *BMP2* is enriched in the pathway of striated muscle tissue development. *ITGA8*, *MYLK*, and *PTCH1* are enriched in the smooth muscle tissue development pathway. *ELOVL1* and *HACD2* are enriched in fatty acid elongation and very long-chain fatty acid biosynthetic process. *BMP2* encodes a secreted ligand of the TGF-*β* protein superfamily, which initiates the classical *BMP* signaling cascade by associating with type I receptor *BMPR1A* and type II receptor *BMPR2* [26]. *BMPR2* phosphorylates and activates *BMPR1A* [27], which in turn relays the phosphorylation signal to the nucleus via *SMAD* proteins. These proteins act as suppressors of target genes. Previous research has shown that proliferation of chondrocytes can regulate osteoclastogenesis via the *BMP2*/*SMAD1*-induced expression of *RANKL* [28]. Studies in chickens have demonstrated that *BMP2* impacts comb quality, egg production, marrow bone area, and the total area and density of bone cavities [29]. Li’s research demonstrated that the SAMD signaling pathway, which involves *BMP2*, plays a role in the proliferation and differentiation of chicken skeletal muscle satellite cells. When *SAMD3* is overexpressed, myostatin secretion increases [30]. Hence, the greater expression of *BMP2* might contribute to the larger body size of CH ducks than of JA ducks. *ITGA8* encodes the alpha 8 subunit of the heterodimeric integrin *α*8*β*1 protein, a transmembrane receptor protein belonging to the alpha integrin family [31]. Pezeshkian et al. reported a strong correlation between the protein encoded by *ITGA8* and chicken growth and feed efficiency, with *ITGA8* being one of the most down-regulated genes in chickens with high feed efficiency [32, 33]. Research on Longyan Shanma ducks revealed that *ITGA8* regulates egg production through the control of actin cytoskeleton formation [34]. *MYLK* encodes myosin light chain kinase, a calcium-dependent enzyme. Malila, Y et al. reported a significant increase in *MYLK* expression in the hypoxic muscles of broiler chickens [35]. *PTCH1* encodes a member of the patched protein family that is located on the plasma membrane and functions as a receptor for sonic hedgehog (SHH), indian hedgehog (IHH), and desert hedgehog (DHH). *PTCH1* mutant mice exhibited impaired glucose tolerance and symptoms of pancreatic malformation in studies [36, 37]. Zhang et al. discovered that *PTCH1* plays a critical role in the development of testes in broiler chickens [38]. In summary, these genes may regulate the larger body size of CH ducks than of JA ducks.

The flavour of meat is strongly linked to its fat composition. *ELOVL1* encodes an enzyme called fatty acid elongase, which plays a significant role in the production of fatty acids and sphingolipids. A study conducted by Castell on rats revealed that *ELOVL1* is involved in the synthesis of very-long-chain (VLC) sphingolipids and contributes to the pancreatic *β* cell proliferation induced by oleic acid [39]. Tanno et al. conducted research on ELOVL isoenzymes and reported that *ELOVL1* plays a role in the production of branched VLC acyl-CoA, which initiates the mitochondrial *β*-oxidation of long-chain fatty acids ranging from 14 to 20 carbons in length [40, 41]. Liu et al. conducted a study on the feeding performance of Xupu geese and Landes wild geese and reported a significant increase in abdominal fat weight in Xupu geese compared to Landes wild geese [42]. This increase was accompanied by a substantial increase in oleic acid content and a significant decrease in *ELOVL1* mRNA expression. According to Liu et al., *ELOVL1* is a member of a gene family responsible for the synthesis of polyunsaturated fatty acids in chickens [43]. Wang et al. demonstrated that the expression of *ELOVL1*–*6* in different chicken organs is closely related to fat deposition and is negatively regulated by estrogen [44].

*HACD2* encodes an enzyme called 3-hydroxyacyl-CoA dehydratase 2, which dehydrates very-long-chain fatty acids during elongation. A study conducted on subcutaneous white adipose tissue revealed lower gene expression of *HACD2* in individuals with lower body weight [45]. Du et al. proposed *HACD2* and *ELOVL5* as potential genes associated with subcutaneous fat deposition in beef cattle [46]. A study on Qinchuan cattle conducted by Yu et al. indicated that *HACD2* is closely related to intramuscular fat deposition [47]. Based on these results, it can be inferred that *ELOVL1* and *HACD2* may play more significant roles in the synthesis and metabolism of fatty acids in CH ducks than in JA ducks. Furthermore, *ELOVL1* may help explain the larger body size observed in male ducks than in female ducks.

CH ducks have predominantly brown plumage, while JA ducks have red plumage. KEGG annotation suggested enrichment of *ASIP* in the pigment deposition pathway and enrichment of *LOC101797494* in the pathway of cytochrome P450 metabolism of xenobiotics. *LOC101797494* encodes cytochrome P450 1B1, named by the International Commission on Cytochrome P450 Nomenclature. *ASIP* encodes the Agouti signaling protein, which acts as an antagonist of the melanocortin-1 receptor (*MC1R*) and plays a role in pigment synthesis [48]. Studies on Japanese quails have shown that *ASIP* leads to decreased melanin pigmentation in feathers [49, 50]. In research on Putian ducks and Longsheng ducks, *ASIP* expression was found only in Longsheng ducks, indicating potential differences in melanin regulation between these species [51]. Cui et al. discovered that *ASIP* exhibits a more ancient mutation than *MC1R* in Monarcha (castaneiventris), potentially explaining the variations in melanin regulation among bird species [52]. The protein encoded by *LOC101797494* contributes to the formation of the cytochrome P450 1B1 enzyme, which is essential for maintaining normal cellular and tissue functions and is a heme-containing monooxygenase [53]. Previous studies suggest that birds produce red pigments through the endogenous conversion of yellow carotenoids to red carotenoids via oxidation catalyzed by ketolase enzymes upon ingestion. An investigation of the *Eurasian tree sparrow* indicated that cytochrome P450 enzymes might play a role in red pigment production in birds [54]. This could account for the difference in body feather color between CH and JA. Additionally, cytochrome P450 enzymes may offer protection against harmful endogenous and environmental compounds, including reactive oxygen species [55].

To the best of our knowledge, while validating genes associated with muscle growth, fat deposition, and pigment deposition in previous studies on domestic ducks, we have also identified genes that may impact the stress resistance and pigment deposition of indigenous ducks in China. Further mechanistic studies are necessary to confirm our findings. Nonetheless, our work contributes to a greater understanding of the genetic factors related to growth benefits, stress resistance, and pigment deposition in the genomic context of Chinese indigenous ducks.

## Conclusion

This study conducted a comprehensive analysis of the population structure and genetic diversity of two local duck breeds on the Middle-lower Yangtze Plain of China, from a genome-wide perspective. Various methods using genomic data were used to assess the inter- and intra-population characteristics of these breeds. The analyses revealed that both breeds were independent populations, with artificial selection currently driving variation in muscle growth, fat deposition, and pigmentation. Additionally, several genes were strongly associated with the expression of economic traits in the breeds. As such, the population structure and candidate genes described in this paper have significant implications for understanding the differentiation of local ducks in southeastern China, improving their breeding programs, managing genetic resources, preserving genetic diversity, and promoting local breeds.

## Materials and methods

### DNA extraction and RAD sequencing

Chaohu and Ji’an Red ducks were raised on a conservation farm at the National Institute of Waterfowl Gene Bank (Shishi, China). Blood samples were collected from the wing veins of 30 CHs and 30 JAs. The ducks represented three generations without a common ancestor, including the existing genetic relationships of each breed. All animal procedures were approved by the Experimental Animal Care and Use Committee of Fujian Agriculture and Forestry University (PZCASFAFU23079) according to the Regulations for the Administration of Affairs Concerning Experimental Animals (Ministry of Science and Technology, China, revised in July 2013).

Genomic DNA was extracted from the blood samples using a Blood Genomic DNA Extraction Kit (Tiangen Biotech Co., Ltd., Beijing, China) following the manufacturer’s instructions. The concentration and integrity of the DNA were assessed using a NanoDrop 2000 (Thermo Fisher Scientific, Leicester, UK) and 1% agarose gel electrophoresis. The DNA libraries of RAD-seq were prepared by Gene Denovo Biotechnology Co., Ltd. (Guangzhou, China) and sequenced on an Illumina HiSeq™ 2500 (Illumina, San Diego, CA, USA) using 150 bp paired-end reads.

### Variant calling

After obtaining the original reads, fastp was used to screen high-quality clean reads. The screening process followed three stringent filtering standards: (1) removing reads aligned to the barcode adapters; (2) removing reads with *>* 50% bases with Phred quality scores of ≤20; and (3) removing reads with ≥10% unidentified nucleotides (N). Picard mark duplicates (https://broadinstitute.github.io/picard/, v2.18.24) were used to remove the PCR-duplicate reads. The clean reads were then mapped to the *Anas platyrhynchos* reference genome (GCF_015476345.1). The Burrows–Wheeler Aligner (BWA) v0.7.17 with default settings was used to obtain sorted binary bam files, which were generated via SAMtools v1.9 [56, 57]. Variant calling for all samples was performed using GATK’s Unified Genotyper (v3.5). Gvcf files were generated by HaplotypeCaller module, and joint genotypes were determined using the GenotypeGVCFs module [58]. GATK’s Variant Filtration was utilized to filter SNPs based on the following criteria: -window 4, −filter “QD *<* 2.0 || FS *>* 60.0 || MQ *<* 40.0”, −G filter “GQ *<* 20”. The minor alleles frequency (maf) and missing genotype rate (geno) were calculated using PLINK (v1.9, Boston, MA, USA) with the parameters “--maf 0.05 --geno 0.2 --allow-extra-chr --chr-set 33”. The “--allow-extra-chr” and “--chr-set” parameters were included to accommodate nonhuman organisms processed by PLINK [59].

### Population structure analysis

A distance matrix was constructed to represent the evolutionary distance between two breeds based on the obtained SNP information. Using the continuous merging method, the samples were cluster analyzed to infer the genetic relationships between populations. MEGA11 software and NJ method were used to construct the phylogenetic tree, which was then visualized using the R package ggtree [60–62]. The SmartPCA program in EIGENSOFT software was used to perform PCA based on SNPs [63]. Population structure analysis was conducted using ADMIXTURE (v1.3.0) software with kinship (K) set from 1 to 9 to observe the Hardy–Weinberg equilibrium [64]. An algorithmic Bayesian model-based analysis was used to calculate the possibility that the genomic variation of each sample originated from the Kth subpopulation, thus inferring the population structure of the samples.

### ROH analysis

ROH were defined as long and continuous homozygous extensions in the genome composed of two identical haplotypes in individuals. PLINK was utilized to estimate the ROH. The minimum number of SNPs required to constitute an ROH was set at 30, with a minimum SNP density of at least 1 SNP per 100 kb per ROH. Additionally, the minimum length of an ROH was set to 300 kb, and the presence of up to two missing and one possible heterozygous genotype was allowed within an ROH. The maximum spacing between consecutive pure heterozygous SNPs was set at 1000 kb. The individual genome inbreeding coefficients (F_ROH_) were calculated by dividing the total length of the duck autosomal genome covered by the SNP by the aggregate length of all detected ROHs in an individual. The top 1% of SNP regions were considered ROH islands after sorting the calculated percentages. The distribution of ROH on these chromosomes in the two indigenous duck populations was mapped using the R package rMVP [65].

### Selective sweep analysis

To identify the genome selection signatures, we employed Fst and XP-CLR calculation methods. The CH and JA samples were categorized together for analysis. Each chromosome was divided into 100 kb windows, and the values of each window on every chromosome were calculated independently. The first window began at the first base, and the starting base position of each subsequent window was positioned 10 kb from the starting base of the previous window. The SNPs contained within the windows were used for Fst and XP-CLR calculations. Fst was determined using the R package PopGenome, while XP-CLR was calculated using XP-CLR software [21, 66]. This approach facilitated the detection of genetic differentiation between CH and JA by establishing a hypothetical selection region for Fst calculations. Considering previous research findings [14, 25], we regarded the top 5% of the results as the selected region and extracted genes within this region. Notably, Fst relies on genomic unit point scanning, which can introduce uncertainty due to genetic drift and other variables, resulting in a lower reliability of the population history factor. Based on previous research [17, 25, 66] and as a supplement to the present study, the top 1% of the XP-CLR index was used as the selected region, as this amplifies the selection signal. Genes within this region were also extracted for further examination. To explore the functional implications of the extracted genes, we conducted GO function and KEGG pathway enrichment analyses. The resulting data were visualized using the R package ggplot2 [67–69]. For these analyses, we utilized the WebGestalt website in conjunction with the David database, and the Genecards website, as well as any available relevant literature, were consulted as references [70–72].

### Supplementary Information

**Supplementary Material 1.**

**Supplementary Material 2.**

**Supplementary Material 3.**

**Supplementary Material 4.**

**Supplementary Material 5.**

**Supplementary Material 6.**

## Data Availability

The dataset supporting the conclusions of this article is available with links to BioProject accession number PRJNA1017640.
