# Supplementary material for: Whole-genome selection signature differences between Chaohu and Ji’an red ducks
Source: BMC Genomics. 2024 May 27;25:522. doi: 10.1186/s12864-024-10339-6 (PMC11131323; doi:10.1186/s12864-024-10339-6)
Supplement: Supplementary file 6 — Supplementary Material 6. [file 12864_2024_10339_MOESM6_ESM.docx]

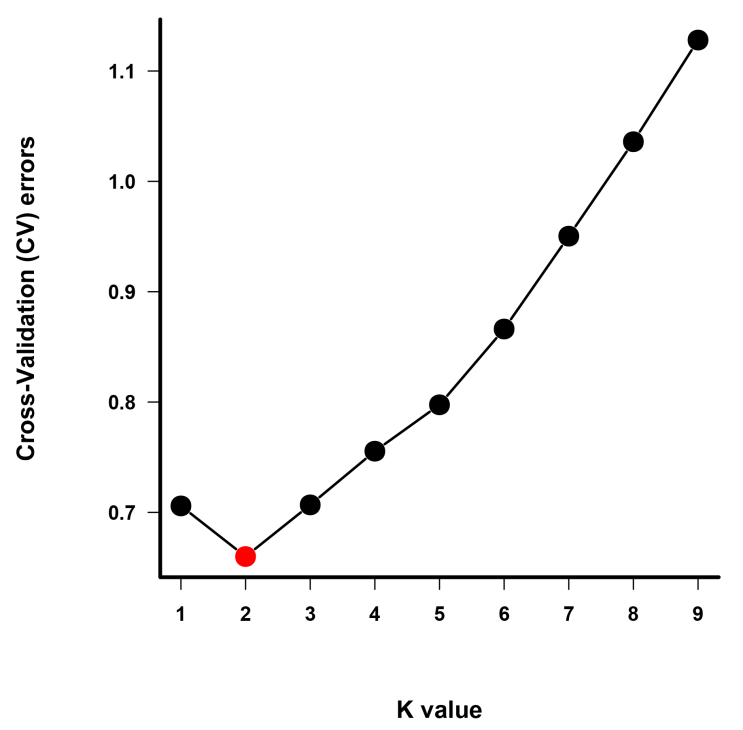


Figure S1. Cross-validation error line charts of duck breeds. The optimal number of assumed ancestors was two (K=2), at which point the error reached the lowest level.


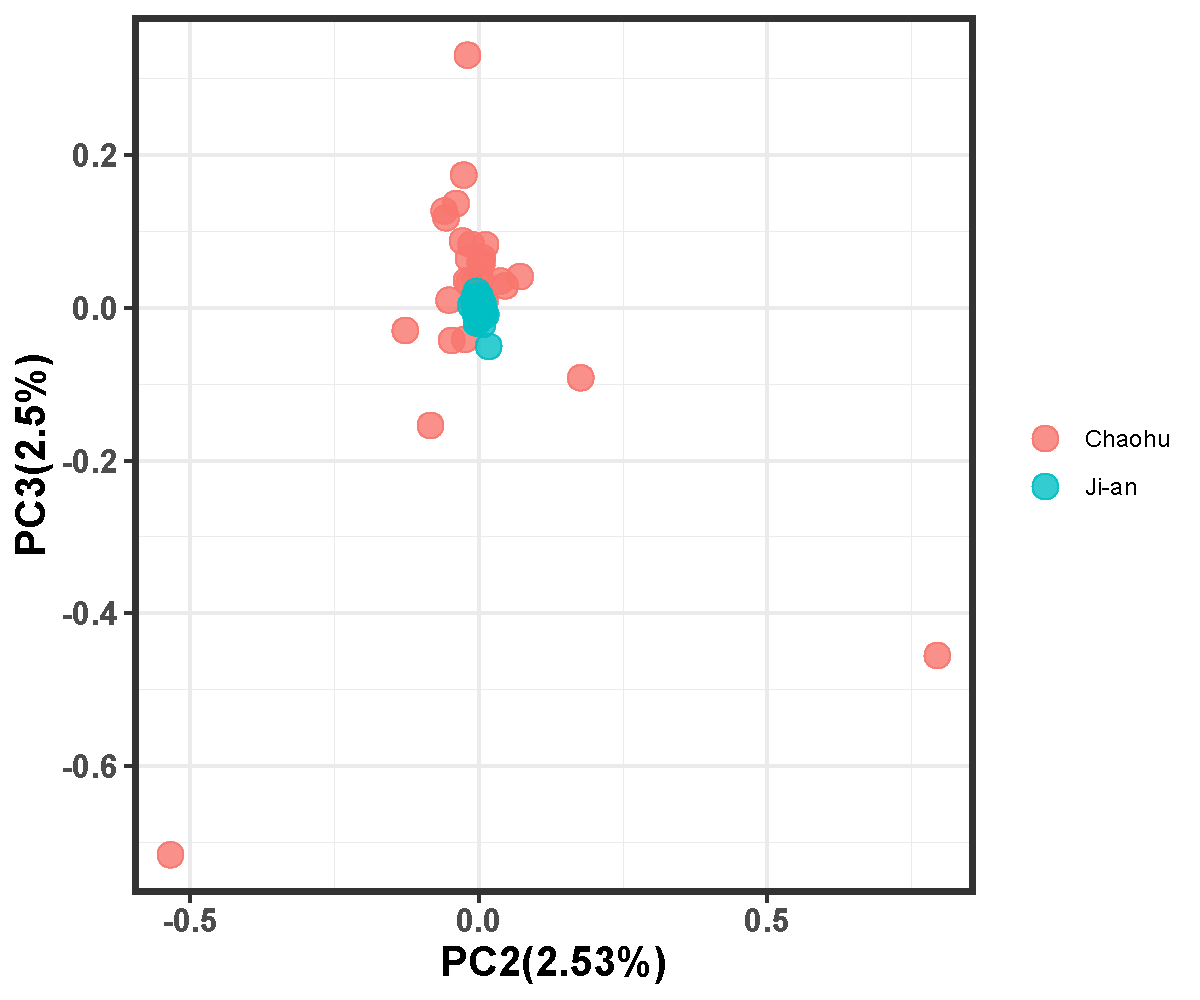


Figure S2. Principal component analysis (PCA) for the second and third component.


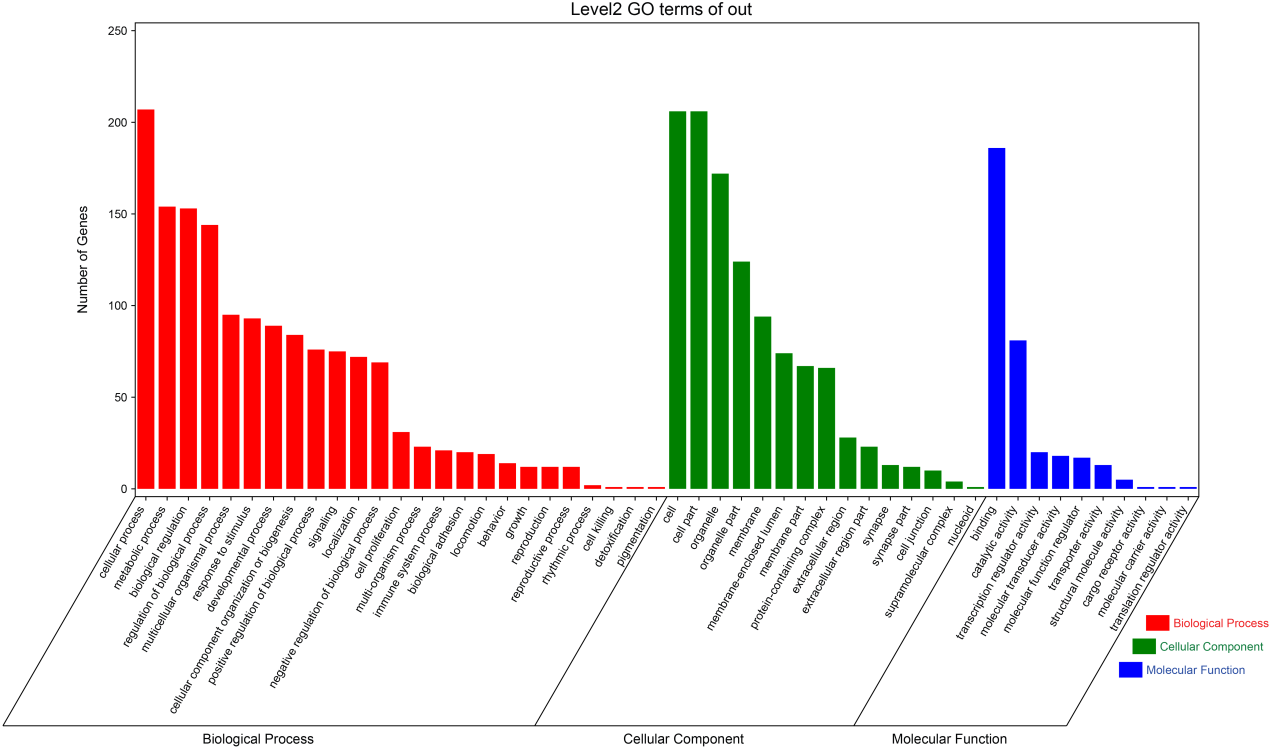


Figure S3. GO enrichment analysis of candidate genes from Fst and XP-CLR screening. The red, green, and blue parts represent biological process, cellular component, and molecular function, respectively.


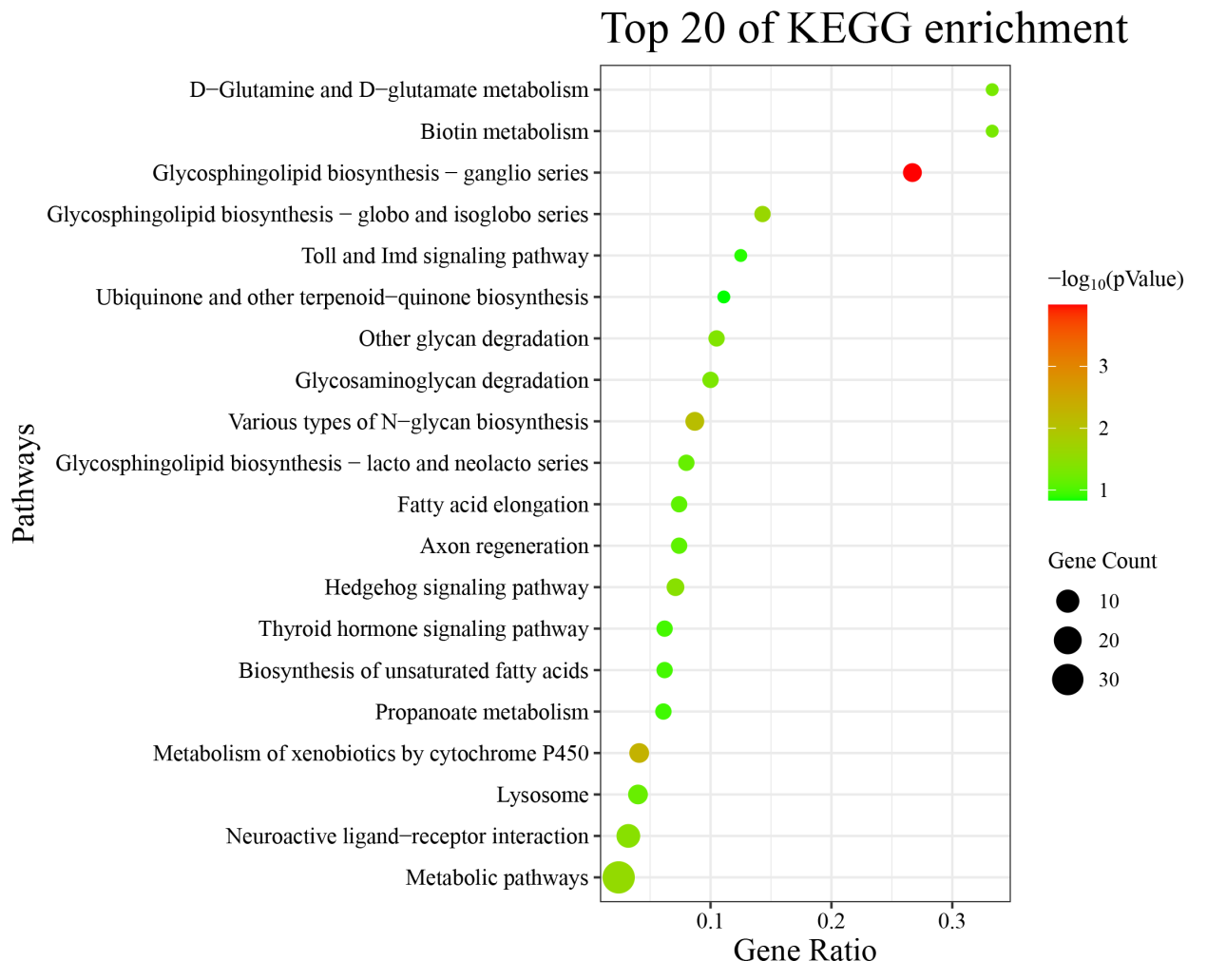


Figure S4. KEGG pathway enrichment analysis of candidate genes from FST and XP-CLR screening.
